# Fixel-Based Analysis Reveals Detailed White Matter Changes in Semantic Dementia

**DOI:** 10.21203/rs.3.rs-6874132/v1

**Published:** 2025-06-16

**Authors:** Maria Luisa Mandelli, Yann Cobigo, Ilaria Perretti, Dana Leichter, Celina Alba, Rian Bogley, Nick Wellman, Siddarth Ramkrishnan, Zachary A. Miller, Bruce L. Miller, William W. Seeley, Howard J. Rosen, Maria Luisa Gorno-Tempini

**Affiliations:** University of California San Francisco; University of California San Francisco; University of Pavia; University of California San Francisco; University of California San Francisco; University of California San Francisco; University of California San Francisco; University of California San Francisco; University of California San Francisco; University of California San Francisco; University of California San Francisco; University of California San Francisco; University of California San Francisco

**Keywords:** fixel-based analysis, diffusion brain imaging, tract-based spatial statistics, white matter changes, semantic dementia, anterior temporal lobe, anterior commissure

## Abstract

**Background and Purpose:**

Accurately characterizing white matter (WM) microstructure is critical for understanding neurodegenerative diseases such as semantic dementia (SD). Regionally constrained techniques like tract-based spatial statistics (TBSS) rely on diffusion-tensor imaging (DTI) and assume a single fiber population per voxel, limiting their sensitivity to complex architecture. Fixel-based morphometry (FBM) overcomes this by assessing multiple fiber populations (fixels) within a single voxel. In this study, we compared TBSS and Fixel-based analysis (FBA) for detecting WM alterations in SD variants associated with anterior temporal lobe (ATL) atrophy.

**Methods:**

Multi-shell diffusion MRI from 16 left-lateralized semantic-variant PPA (svPPA) and 15 right-lateralized semantic-behavioral fronto-temporal dementia (sbvFTD) cases, plus 44 neurologically healthy controls, underwent both TBSS-DTI and whole-brain FBA. Fiber-specific metrics of fiber density and cross-section were contrasted with conventional DTI measures.

**Results:**

Both methods confirmed damage to ATL-connected tracts—the uncinate fasciculus, inferior longitudinal fasciculus, inferior fronto-occipital fasciculus, and temporal projections of the arcuate fasciculus. FBA, however, revealed additional involvement of juxtacortical and other previously overlooked pathways, including the tapetum and anterior commissure, projections to the parahippocampal gyrus and amygdala, and longer-range parietal connections.

**Conclusions:**

By capturing fiber-specific micro- and macrostructural changes, FBA yields a more comprehensive map of WM degeneration in SD than TBSS. The ability to detect early alterations in commissural and mesial-temporal pathways refines our understanding of disease spread and highlights candidate targets for monitoring and intervention aimed at preserving cognitive function.

## INTRODUCTION

Over the past two decades, diffusion MRI (dMRI) has significantly advanced our ability to study white matter (WM) microstructure, enabling non-invasive, in-vivo visualization of fiber pathways and their organization. By quantifying WM integrity, dMRI provides critical insights into neurodegenerative disease progression, revealing subtle microstructural alterations often undetectable by conventional MRI (Goveas et al. 2015). These findings underscore the role of WM connectivity disruptions in conditions such as Alzheimer’s disease, mild cognitive impairment, amyotrophic lateral sclerosis (Li et al. 2012; Oishi et al. 2011), and frontotemporal dementia (Federica Agosta et al. 2010; Galantucci et al. 2011; Whitwell et al. 2010), supporting the conceptualization of neurodegenerative disorders as network-level disconnection syndromes (F. Agosta et al. 2012; Fletcher and Warren 2011). However, conventional dMRI methods, such as diffusion tensor imaging (DTI), are limited in capturing the complexity of WM fiber geometry, reducing biological specificity and challenging interpretation (Basser, Mattiello, and LeBihan 1994; Jeurissen et al. 2019; Pierpaoli et al. 2001; Wheeler-Kingshott and Cercignani 2009). Metrics like fractional anisotropy (FA) and mean diffusivity (MD), typically analyzed using voxel-based approaches or tract-based spatial statistics (TBSS) (Smith et al. 2006), average diffusion properties within each voxel, obscuring the presence of multiple fiber populations and their unique characteristics. To overcome these limitations, Fixel-based analysis (FBA) has been introduced as an anatomically precise framework for studying WM alterations (Raffelt et al. 2015, 2017). Unlike conventional approaches, FBA differentiates distinct fiber populations (‘fixels’) within each voxel, allowing fiber-specific quantification of micro- and macrostructural changes. Furthermore, FBA facilitates a whole-brain approach without restricting measurements to the white matter “skeleton,” as required by TBSS. This approach is particularly well-suited for detecting subtle changes in juxtacortical regions and capturing distributed, network-level processes underlying neurodegenerative diseases.

Semantic-variant primary progressive aphasia (svPPA) (Gorno-Tempini et al. 2011), also known as semantic dementia (SD) (Hodges et al. 1992; Snowden, Goulding, and Neary 1989), is a clinical syndrome within the frontotemporal dementia spectrum characterized by focal, often asymmetric, atrophy of the anterior temporal lobes (ATLs). Degenerative changes typically involve the temporal poles, medial temporal limbic structures (including the hippocampi and amygdalae), anterior lateral temporal neocortical areas, and posterior insulae (Binney et al. 2016; Bocchetta et al. 2020; Borghesani et al. 2020; Chan et al. 2001; Ding et al. 2016). These regions form broader cortical and subcortical networks supporting semantic memory, and socio-emotional processing (Benhamou et al. 2020; Fletcher and Warren 2011; Guo et al. 2013; Seeley 2008), highlighting the importance of investigating connectivity within these neural circuits.

FBA offers a robust framework for assessing structural connectivity in SD by analyzing distinct fiber populations within each voxel. This enhanced specificity enables detection of subtle alterations in small, intricate pathways – such as the anterior commissure – that conventional DTI might overlook. By accurately capturing complex neuroanatomical configurations, FBA yields deeper insights into network-level disruptions associated with clinical manifestations of SD. Although svPPA (and semantic dementia) has historically been associated with left-predominant ATL atrophy (Chan et al. 2001; Hodges et al. 1992; Mummery et al. 2000; Snowden, Goulding, and Neary 1989), recent studies suggest that at least one-third of cases show right-predominant ATL degeneration (Borghesani et al. 2020; Chan et al. 2009; Edwards-Lee et al. 1997; Gainotti, Barbier, and Marra 2003; Hodges et al. 2004; Kumfor et al. 2016; Patterson, Nestor, and Rogers 2007; Snowden et al. 2018). These cases have recently been classified as semantic behavioral variant frontotemporal dementia (sbvFTD) (Younes et al. 2022), or right temporal FTD. Here, we use “semantic dementia” as an umbrella term encompassing both svPPA and sbvFTD. Regardless of initial lateralization, the disease eventually progresses contralaterally and extends to orbitofrontal, anterior cingulate, and temporoparietal regions (Borghesani et al. 2020; Brambati et al. 2009; Josephs et al. 2011; Kumfor et al. 2016; Rohrer et al. 2009; Spinelli et al. 2017). Investigating both left- and right-sided pathways is therefore crucial for capturing the full disease spectrum.

DTI studies on SD have consistently highlighted involvement of major WM tracts connecting the ATL to other cortical regions, including the inferior longitudinal, arcuate, and uncinate fasciculi, while showing relative sparing of dorsal frontoparietal pathways (Acosta-Cabronero et al. 2011; Federica Agosta et al. 2010; Whitwell et al. 2010). However, interhemispheric connections - such as the anterior commissure or the tapetum of the corpus callosum (CC) - have received less attention, likely due to their small size and complex configuration, complicating delineation with conventional DTI approaches. Detailed examination of these pathways could significantly enhance understanding of WM microstructural alterations across hemispheres and provide new insights into network-driven degeneration underlying SD.

In this study, we assessed WM integrity in SD using FBA and TBSS-DTI applied to high-angular-resolution, multi-shell MR diffusion images. Our sample included 16 individuals with svPPA, 15 individuals with sbvFTD, and a control group of 44 healthy control participants. We hypothesized that FBA would demonstrate greater sensitivity compared to the conventional DTI approaches in detecting WM changes in intra- and interhemispheric pathways connecting the ATL and related regions, thereby enhancing our understanding of fiber-specific disruptions underlying SD.

## METHODS

### Participants

Patients with a clinical diagnosis of svPPA or sbvFTD were recruited from the database available at the Memory and Aging Center, University of California, San Francisco (MAC-UCSF). To focus the analysis on the earlier phase of the disease, patients were included if at the time of the imaging their Mini-Mental State Examination (MMSE) was greater than or equal to 14, and their Clinical Dementia Rating (CDR) was less than or equal to 2. Participants were selected based on the availability of a multi-shell diffusion-weighted imaging sequence acquired, which enables advanced fiber orientation modelling required for FBA and improved tract-specific estimation (Jeurissen et al. 2014). We divided the patients into right- or left- predominant ATL atrophy based on a lateralization index derived from structural brain MRI, as detailed below. Neurologically healthy, community-dwelling older adults were recruited from a cohort enrolled in the BRain Aging Network for Cognitive Health (BRANCH) study at the MAC-UCSF. This cohort includes in-person behavioral and neuroimaging measurements. Participants were verified as neurologically healthy based on a multidisciplinary assessment, including neurological examination, neuropsychological testing, and cognitive assessment (Kramer et al. 2003).

### Brain MRI acquisition

MRI scans were collected at the UCSF Neuroscience Imaging Center on a 3T Siemens Magnetom Prisma Scanner. Magnetization-prepared rapid acquisition with gradient echo acquisition (MPRAGE) was used to acquire T1-weighted images with the following parameters: 160 sagittal slices, isotropic voxel size = 1mm^3^, repetition time (TR)/echo time (TE) = 2300/2.98 ms, inversion time = 900 ms, flip angle = 9°, field of view (FoV) read of 256×256 mm^2^, and an integrated parallel acquisition technique (iPAT) acceleration factor of 2. Diffusion-weighted imaging sequences were collected with a single-shot spin-echo echo-planar imaging (SE-EPI) sequence with the following parameters: 69 contiguous axial slices with in-plane resolution = 2×2mm^2^, TR/TE = 2420/72.20ms, flip angle = 85°, FoV of 220×220mm^2^, 96 non-collinear diffusion sensitization directions at b = 2500 s/mm^2^, 48 directions at b = 1000 s/mm^2^, 30 directions at b = 500 s/mm^2^, and 10 volumes at b = 0, with an iPAT acceleration factor of 2 and multi-band acceleration factor of 3. Two additional b = 0 volumes were acquired with opposite phase-encoding directions (anterior/posterior and posterior/anterior) for distortion correction. Visual quality inspections were conducted for all the MR images, and any images with anomalies, artifacts, excessive motion, or significant white matter hyperintensity were excluded.

### Structural brain processing

#### Temporal atrophy index for group assignment

This study includes participants with moderate and focal atrophy predominantly affecting either the left or right temporal regions. We calculated an atrophy index reflecting the lateralization of temporal atrophy and the proportion relative to frontal lobe involvement, following a similar procedure as previously described (Younes et al. 2022). We extracted the cortical volume within the temporal and frontal lobes using the Desikan atlas as a reference (Desikan et al. 2006) after processing the structural images (Ashburner and Friston 2005, 2011; Manjón et al. 2010; Rajapakse, Giedd, and Rapoport 1997) through the Computational Anatomy Toolbox within the framework of Statistical Parametric Mapping software (SPM12; fil.ion.ucl.ac.uk/spm/software/spm12). Specifically, the temporal regions consisted of the temporal pole, entorhinal cortex, fusiform gyrus, banks of the superior temporal sulcus, transverse temporal gyrus, and inferior, middle, and superior temporal gyri. The frontal brain regions included the inferior, middle, and superior frontal gyri. For each anatomical region, volume W-scores were generated from normative data derived from a control group of healthy individuals, adjusted for age and sex. W-scores are standardized with a mean of 0 and a standard deviation of 1; scores of + 1.65 and − 1.65 denote the 95th and 5th percentiles, respectively, indicating cortical volumes that are significantly larger or smaller compared to the normative cohort. Patients were considered to be in the left ATL-predominant atrophy group if the highest W-score was in the left temporal region with a ratio of the mean frontal W-score to the mean left temporal W-score of less than 0.50. Similarly, patients were included in the right ATL-predominant atrophy group if the highest W-score was in the right temporal regions with a mean frontal W score to mean right temporal W-score ratio of less than 0.50. This stratification ensures the selection of patients who have relatively intact frontal lobes, thus likely representing those who are at an earlier stage of the disease from an anatomical perspective.

### Diffusion-weighted imaging analysis

#### Preprocessing

The diffusion data were pre-processed using the FMRIB Software Library (FSL) (Smith et al. 2004). Susceptibility-induced off-resonance fields were estimated from two volumes acquired in opposite phase-encoding directions using the FSL ‘topup’ tool and eddy current distortions and head motion were corrected using the FSL ‘eddy’ tool ((Andersson, Skare, and Ashburner 2003; Andersson and Sotiropoulos 2016).

#### Diffusion tensor imaging and TBSS

Tensor-based registration was used to generate single-subject tensor maps aligned to a common template (Keihaninejad et al. 2012; Zhang et al. 2007). An inter-subject group template was generated through iterative linear and non-linear registrations of the diffusion tensor images. Four diffusion metrics FA, MD, radial diffusivity (RD), axial diffusivity (AD) - were derived from the fitted tensors for each subject. Visual quality inspection was conducted to check for any registration errors. Analysis of the DTI metrics was performed using tract-based spatial statistics (TBSS). The FA maps for each subject were averaged to generate a single mean FA map, which was thinned with a threshold of 0.2 to create a skeletonized FA image representing the center of the WM tracts common to all participants (Smith et al. 2006). The aligned FA, MD, RD, and AD maps for each subject were projected onto this FA skeleton. The resulting skeletonized maps were used in voxel-wise cross-subject statistical analysis, using FSL *randomise* with 5000 permutations (Winkler et al. 2014). The two cohorts of patients (svPPA and sbvFTD) were compared respectively to the healthy control group. All models were controlled for the potential confounding effects of age, sex, and total intracranial volume (TIV). P-values were corrected with threshold-free cluster enhancement, and P < 0.05 was set as the significance threshold (Smith and Nichols 2009). Anatomical localization of the significant results along the main tracts was performed using the HCP1065 Population-Averaged Tractography Atlas (Yeh 2022). The Supplementary Material presents a color-coded map of the white-matter tracts described in this study; the same color scheme and section references are used in Figs. 2 and 3.

### Fixel-based morphometry

Fixel-based morphometry was performed using the MRtrix3 toolbox (Raffelt et al. 2017; Tournier et al. 2019). The diffusion images were first pre-processed and upsampled to a resolution of 1.25mm. We applied a multi-shell, multi-tissue Constrained Spherical Deconvolution (MSMT-CSD) (Jeurissen et al. 2014; Tournier et al. 2004; Tournier, Calamante, and Connelly 2007) to the denoised (Veraart et al. 2016) and unringed (Kellner et al. 2016) diffusion images to extract the Fiber Orientation Distribution Functions (FODs). Bias field correction and global intensity normalization were applied to the individual subject FODs, and an unbiased study specific FOD template was created using the entire cohort. All individual subject FODs were registered to the template space and segmented to extract the fixels within each voxel. Fixel Density (FD) is a micro-structural measure computed from the FOD. For each voxel, the FOD is examined to locate its distinct lobes—each lobe representing a dominant fiber orientation, or fixel. The amplitude of a given lobe is then integrated along its direction, yielding FD. Consequently, FD estimates the intra-axonal volume of that specific fiber population, effectively capturing how much axonal material is aligned in that direction. Fixel Cross-Section (FC) is a macro-structural metric derived from the orthogonal scaling component of the deformation field; it quantifies how the cross-sectional area of a given fiber bundle must change to match a population template, thereby capturing differences in white-matter bundle morphology across individuals or groups. Their combined measure (FDC, Fixel Density Cross-Section) was computed as the product of FD and FC (Dhollander et al. 2021) integrating the information on microstructural integrity and tract atrophy, offering a more comprehensive assessment of disease-related WM damage. Statistical comparisons of FD, FC, and FDC between groups were performed at each fixel by a general linear model, comparing (i) svPPA versus healthy controls and (ii) sbvFTD versus healthy controls. Sex and age were included as covariates in the model. Statistical significance was determined using a family-wise error (FWE)-corrected threshold of p < 0.05.

Connectivity-based smoothing and statistical inference was performed using connectivity-based fixel enhancement, using 2 million streamlines from the template tractogram, and with default smoothing parameters (smoothing = 10 mm full-width at half-maximum, C = 0.5, E = 2, H = 3) (Raffelt et al., 2015). FWE-corrected P-values were then assigned to each fixel using non-parametric permutation testing over 5000 permutations (Nichols and Holmes 2002).

## RESULTS

### MRI data quality control

The inclusion criteria required an MMSE score greater than or equal to 14, no motion or other MRI artifacts, and no evidence of white matter hyperintensity (Fazekas > 2). Of the 92 scans selected for this study (n = 23 svPPA, n = 25 sbvFTD, n = 44 healthy controls), several did not meet these inclusion criteria or failed the image quality control and were subsequently excluded from the processing and analysis. Ultimately, 17 scans were excluded, leaving a final sample of 75 participants: 16 svPPA, 15 sbvFTD, and 44 healthy controls. A summary of the demographic and severity profiles across our groups of interest is presented in Table 1.

### Gray matter analysis

**Figure 1:**
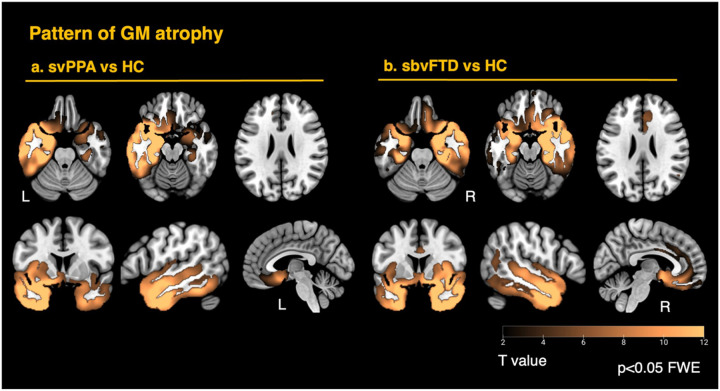
Gray-matter atrophy in the two anterior-temporal variants Voxel-based morphometry maps illustrate convergent atrophy in the temporal pole, amygdala– hippocampal complex, and ventral limbic structures in both patient groups. (a) Semantic-variant primary progressive aphasia (svPPA) shows left-dominant loss; (b) semantic-behavioral variant frontotemporal dementia (sbvFTD) shows the right-lateralized mirror pattern with additional anterior cingulate cortex involvement. Warm colors indicate increasing t-values (greater atrophy relative to the healthy controls group). Statistical maps were family-wise-error (FWE) corrected at p < 0.05)

Gray matter atrophy patterns confirmed predominant involvement in temporal and limbic regions, showing asymmetrical patterns between the two groups of patients (Fig. 1). The overall atrophy patterns were largely mirrored between the two variants, though the sbvFTD group exhibited slightly greater gray matter loss, particularly in the anterior cingulate. This difference may be attributed to the tendency of individuals with predominant right ATL involvement - who often present with behavioral symptoms - to seek clinical evaluation at a later stage compared to those with predominant left ATL involvement, who are more likely to be diagnosed earlier due to semantic language deficits.

### TBSS analysis of DTI metrics

#### Differences in svPPA versus healthy controls

**Figure 2:**
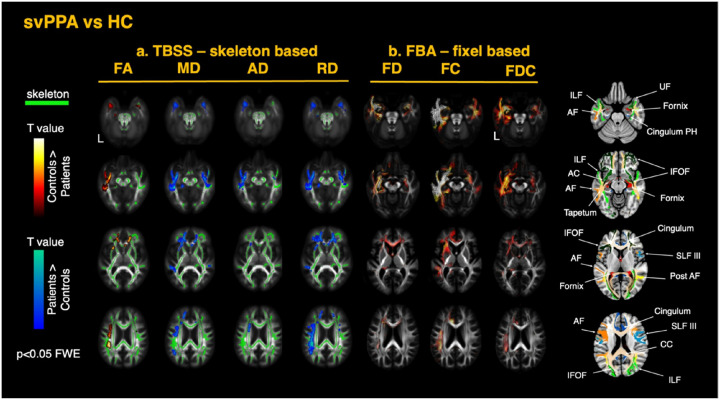
White matter alterations in semantic variant PPA (svPPA) relative to healthy controls (a) TBSS—skeleton-based analysis: group contrasts of diffusion-tensor–derived metrics—fractional anisotropy (FA), mean diffusivity (MD), axial diffusivity (AD), and radial diffusivity (RD)—are shown on the TBSS skeleton (green) overlaid on the MNI-152 template. (b) FBA— fixel-based analysis: corresponding contrasts for fiber density (FD), fiber cross-section (FC), and the combined metric fiber density-and-cross-section (FDC). Statistical maps are thresholded at family-wise-error (FWE)–corrected p< 0.05. Warm colors (white → red) indicate greater metric values in controls than patients, whereas cool colours (cyan → blue) indicate lower metric values in controls than patients. On the right panels, axial slices highlight the principal tracts exhibiting significant change, including the uncinate fasciculus (UF), inferior longitudinal fasciculus (ILF), inferior fronto-occipital fasciculus (IFOF), arcuate fasciculus (AF), tapetum, anterior commissure (AC), fornix, parahippocampal cingulum, superior longitudinal fasciculus (SLF), posterior thalamic radiation, and corpus callosum (CC).

In the svPPA group, TBSS analysis revealed significant changes in the DTI metrics with decreased FA and increased diffusivity metrics (MD, AD, RD) primarily in the supratentorial WM tracts connected to the left ATL, including the inferior longitudinal fasciculus (ILF), the uncinate fasciculus (UF) and lateral portions of the inferior frontal occipital fasciculus (IFOF). Other WM structures also showed significant involvement, including the temporal and parietal projections of the left arcuate fasciculus (AF), the left external capsule, and key limbic system structures such as the fornix and left parahippocampal gyrus. Additionally, significant changes were noted in the genu of the CC, and bilaterally in the anterior corona radiata. A summary of the TBSS findings for svPPA is displayed in Fig. 2A. Overall, FA showed the most differences primarily in the affected hemisphere, whereas increased MD was more bilateral in the ventral tracts. RD was more sensitive than AD, primarily driving the observed differences in MD. This finding supports previous studies suggesting that RD is the most sensitive DTI measure for detecting pathological changes (Galantucci et al 2011; Acosta-Cabronero et al 2011; Agosta et al 2012; Agosta et al 2015).

#### Differences in sbvFTD versus healthy controls

**Figure 3:**
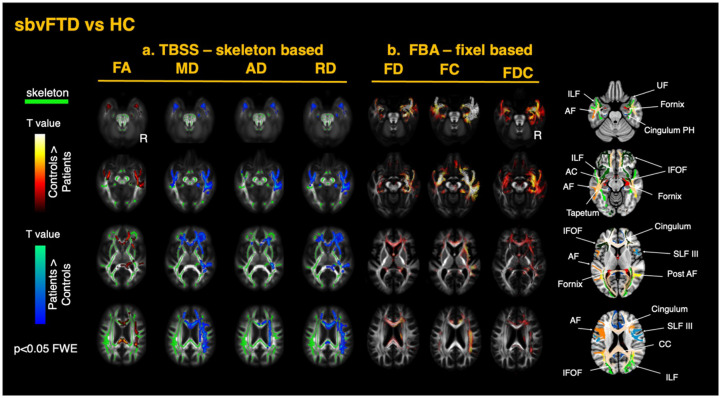
White matter alterations in semantic behavioral variant frontotemporal dementia (sbvFTD) relative to healthy controls (a) TBSS—skeleton-based analysis: group contrasts of diffusion-tensor–derived metrics—fractional anisotropy (FA), mean diffusivity (MD), axial diffusivity (AD), and radial diffusivity (RD)—are shown on the TBSS skeleton (green) overlaid on the MNI-152 template. (b) FBA2014 fixel-based analysis: corresponding contrasts for fiber density (FD), fiber cross-section (FC), and the combined metric fiber density-and-cross-section (FDC). Statistical maps are thresholded at family-wise-error (FWE)–corrected p < 0.05. Warm colors (white → red) indicate greater metric values in controls than patients, whereas cool colors (cyan → blue) indicate lower metric values in controls than patients. On the right panels, axial slices highlight the principal tracts exhibiting significant change, including the uncinate fasciculus (UF), inferior longitudinal fasciculus (ILF), inferior fronto-occipital fasciculus (IFOF), arcuate fasciculus (AF), tapetum, anterior commissure (AC), fornix, parahippocampal cingulum, superior longitudinal fasciculus (SLF), posterior thalamic radiation, and corpus callosum (CC).

A similar but mirrored pattern of changes in DTI metrics was observed in the sbvFTD group. Effects were observed in WM tracts connected to the right ATL, including regions of the limbic system, with additional involvement of the body and splenium of the CC, which were not observed in svPPA. This group exhibited more bilateral involvement overall consistent with the gray matter atrophy pattern. A summary of the TBSS findings for the sbvFTD group is displayed in Fig. 3A, showing decreased FA and increased diffusivity metrics across the same structures as in the svPPA group. Similarly, RD emerged as the most sensitive metric. The consistency of these findings across the two groups of patients suggests a common pathophysiological mechanism driving the damage characteristic of the disease.

### Fixel-Based Analysis

#### Differences in svPPA versus healthy controls

**Figure 4:**
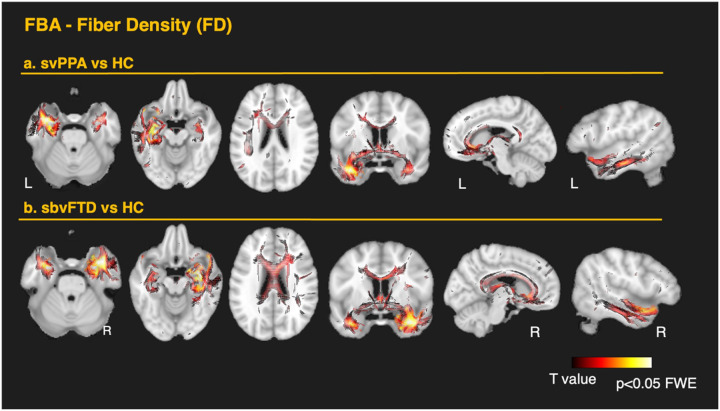
Fixel-based analysis of fiber density loss in the two anterior temporal variants. Whole-brain xel-wise contrasts of fiber density (FD) are shown thresholded at family-wise-error (FWE) corrected p < 0.05 are overlaid on the MNI template. Warm colors (dark red → yellow) denote lower FD in patients relative to the healthy controls group. (a) svPPA vs controls. Pronounced left-lateralized reductions involve the uncinate (UF), inferior longitudinal (ILF) and inferior fronto-occipital fasciculi (IFOF, including orbitofrontal and fusiform projections), temporal projections of the arcuate fasciculus (AF), and the parahippocampal cingulum. Additional losses are noted in the anterior cingulate, in the anterior commissure, tapetum, splenium, and fornix. (b) sbvFTD vs controls. A largely right-hemispheric mirror pattern emerges, but with more extensive bilateral ventral involvement. FD loss affects the ILF, UF, IFOF, temporal AF, parahippocampal cingulum. Additional losses are noted in the anterior cingulate, in the anterior commissure, tapetum, splenium, and fornix. Significant reductions extend throughout the corpus callosum (genu, body, and splenium).

**Figure 5:**
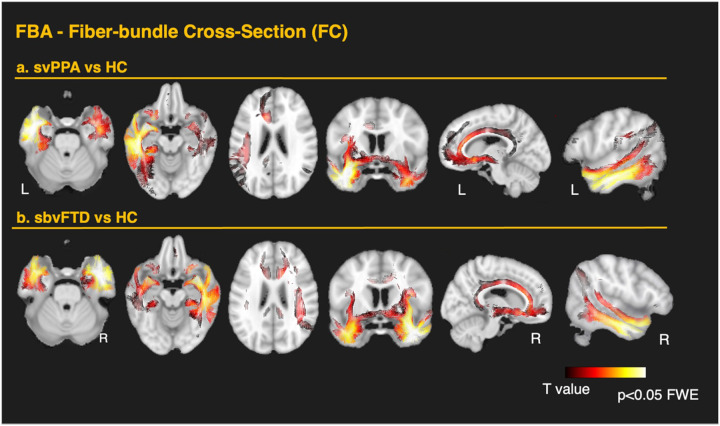
Fixel-based analysis of fiber-bundle cross-section in the two anterior-temporal variants. Whole-brain fixel-wise contrasts of fiber-bundle cross-section (FC) thresholded at family-wise-error FWE corrected p < 0.05 are overlaid on the MNI template. Warm colors denote a reduced FC in patients relative to controls. (a) svPPA vs controls. Marked left-lateralized bundle narrowing spans ventral association tracts (UF, ILF, IFOF), fornix, parahippocampal cingulum, anterior commissure, and tapetal callosal fibers, with extension into the long segment of the AF (parietal projections). FC reductions also encompass the subgenual and mid-cingulate portions of the cingulum, the anterior commissure, tapetum, splenium, and fornix. (b) sbvFTD vs controls. A right-lateralized yet bilaterally extensive pattern appears. FC reduction mirrors ventral temporal pathways and extends dorsally from the temporal pole through inferior temporal cortex to the inferior parietal/angular gyrus. FC reductions are also evident in the anterior–mid cingulum, anterior commissure, tapetum, splenium, and fornix.

In the svPPA group, FBA showed significant reductions of FD, FC, and FDC in the left ILF, UF, IFOF (including projections to the orbitofrontal areas and around the fusiform), in the temporal projections of the AF, in the anterior cingulate, and parahippocampal gyrus. The most significant changes were observed in the affected hemisphere, though some involvement of contralateral ventral structures was also noted. Additionally, changes were observed in the anterior corona radiata, as well as in the internal and external capsules. FDC reductions overall corresponded with the changes observed for FD and FC. Significant changes in the anterior commissure, tapetum, cingulum bundle, and fornix were evident – pathways not captured by the skeleton-based TBSS analysis. A summary of the FBA findings for the svPPA group compared to the TBSS approach is displayed in Fig. 2B. Overall, FC exhibited greater impairment than FD, with the most pronounced effects observed in the WM tracts connecting the ATL and the temporo-mesial structures such as the amygdala and parahippocampal gyrus. More detailed results are shown in Fig. 4A for FD and Fig. 5A for FC. Interestingly, all FBA metrics showed significant decreases in the subgenual segment of the cingulum, while FC and FDC reductions extended from the anterior to the midcingulate area. Conversely, significant reductions in the anterior CC were observed only in FD and FDC, with no changes in FC. These opposing patterns may reflect distinct or secondary pathophysiological mechanisms beyond simple neural loss due to atrophy in these areas.

#### Differences in sbvFTD versus controls

FBA revealed similar changes in the sbvFTD group, mirroring the svPPA group but with more extensive bilateral ventral involvement. A summary of the FBA findings for the sbvFTD group compared to the TBSS approach is displayed in Fig. 3B. The most prominent loss of FD was observed bilaterally in the ILF and UF. Significant reductions were also detected in several parts of the IFOF, in the temporal projections of the AF, in the cingulate projections to the parahippocampal gyrus, the anterior commissure, the tapetum, the fornix, the anterior corona radiata, and both the internal and external capsules. In contrast to svPPA, the sbvFTD group showed significant changes throughout the entire CC. As in svPPA, FC reductions were more pronounced than FD (Fig. 3B). These changes extended dorsally from the temporal pole of the affected hemisphere to the inferior temporal gyrus, fusiform area, and the inferior parietal area, including the angular gyrus. More detailed results are shown in Fig. 4B for FD and Fig. 5B for FC. As in svPPA, FC and FDC reductions, but not FD changes, were detected bilaterally in the anterior cingulum in the sbvFTD group. Similarly, FD and FDC changes, but not FC, were observed throughout the entire CC, including the posterior regions.

## DISCUSSION

This study provides novel insights into WM degeneration in SD by comparing TBSS-DTI with FBA on multi-shell diffusion MRI data. As hypothesized, the fiber-specific precision afforded by FBA revealed subtle WM alterations that TBSS failed to detect. Notably, FBA identified additional disruptions in interhemispheric ATL connections (e.g. anterior commissure), underscoring its heightened sensitivity to small and intricate fiber pathways. These findings highlight the value of advanced diffusion imaging techniques in detecting subtle network disruptions underlying neurodegenerative diseases. In the following sections, we discuss the technical advantages of FBA and the novel anatomical insights it provides regarding ATL connectivity in semantic dementia.

### Comparison of TBSS versus FBA: Advantages and challenges

Diffusion MRI is currently the only in vivo technique capable of characterizing WM fiber architecture, making it a crucial tool for studying connectivity in neurodegenerative diseases. TBSS has been widely used due to its robustness in aligning FA maps across subjects and its ability to reduce partial volume effects by restricting analyses to a WM skeleton. This approach minimizes misregistration errors and allows for reliable voxel-wise statistical comparisons of DTI metrics such as FA and MD (Smith et al. 2006). However, TBSS has well-documented limitations, including its inability to resolve crossing fibers — ubiquitous throughout the brain—and its reliance on projecting diffusion data onto a group-averaged skeleton, which can lead to signal loss in peripheral or anatomically complex regions (Bach et al. 2014; Zalesky 2011). FBA represents a significant methodological advancement by enabling fiber-specific quantification within each voxel (Raffelt et al. 2017). Unlike TBSS, which averages scalar values across all fiber populations within a voxel, FBA disentangles individual fiber populations (fixels), allowing for the estimation of fiber-specific metrics such as FD, FC, and their combined product FDC. These metrics improve sensitivity to both microstructural degeneration and macrostructural atrophy, particularly in regions with complex fiber architecture. In the context of neurodegeneration, FBA has been successfully applied to detect tract-specific changes not captured by conventional DTI approaches (Dewenter et al. 2023; Mito et al. 2018; Oh et al. 2021; Rau et al. 2019; Savard et al. 2022). Notably, Savard et al. (2022) demonstrated widespread reductions in FC and FD across all FTD variants. In svPPA specifically, they reported left-lateralized FC reductions in the ILF—a key ventral pathway—highlighting FBA’s ability to resolve variant-specific degeneration with greater anatomical specificity than standard diffusion approaches. Their findings also support the notion that FC may be more sensitive than FD in capturing the spatial extent of white matter damage, a pattern consistent with our results. In our study, we extended these findings by examining finer grained and under-characterized pathways, including the anterior commissure and projections to mesial temporal and limbic structures. These results illustrate the additional anatomical precision afforded by FBA, particularly in detecting degeneration of small or juxtacortical tracts that may be overlooked by skeleton-based approaches like TBSS.

### Novel insights into interhemispheric ATL connectivity

Our results highlight the anterior commissure and the tapetum as critical conduits for cross-hemispheric spread of pathology in SD. The AC links the anterior-inferior temporal cortices and, as Fletcher and Warren (2011) argued, may provide the structural route through which pathology propagates between temporal lobes in network-level disconnection syndromes. Technical limitations have left only sparse empirical evidence of AC degeneration in SD (Moon et al. 2008). Using FBA, we detected convergent reductions in both FC and FD in the AC, most pronounced in the right-temporal (sbvFTD) group. This mirrors the broader bilateral gray matter involvement in sbvFTD and supports the commissure’s role in inter-hemispheric disease spread. Future studies correlating FBA-derived metrics with behavioral and cognitive profiles will further clarify the functional significance of anterior commissure degeneration in SD. The tapetum, the CC segment that joins the medial temporal lobes, also emerged as a key structure.

FBA revealed reduced FD with relatively preserved FC, consistent with microstructural injury that precedes overt tract atrophy. Longitudinal diffusion MRI work in svPPA has already documented progressive tapetal degeneration (Elahi et al. 2017); our findings indicate that such alterations are detectable at earlier disease stages. Because these microstructural changes precede macroscopic volume loss, tapetal FD may serve as an early biomarker for tracking progression (Downey et al. 2015; Tu et al. 2016).

Although semantic dementia is usually driven by FTLD-TDP type C pathology, how pathological TDP-43 crosses from one hemisphere to the other remains uncertain. Prion-like, trans-synaptic spread is well established for aggregated tau, yet whether TDP-43 follows the same route or employs a different mechanism is still under debate, despite emerging evidence that hints at prion-like behavior (Keszycki et al. 2022). Commissural pathways—such as the anterior commissure, tapetum, and broader anterior CC— are anatomically plausible conduits, but direct proof of TDP-43 trafficking along these tracts is lacking. Combining ultra-high-field diffusion imaging with post-mortem tract-tracing and biochemical studies will be crucial to test this hypothesis and clarify how interhemispheric connectivity influences clinical phenotype and progression in SD.

### Greater definition of mesial-temporal connectivity in semantic dementia

Our results reveal damage to the limbic/temporo-mesial pathways—the UF cingulum bundle, and fornix— that interlink the hippocampus, amygdala, anterior thalamus, and orbitofrontal cortex (Catani, Dell’Acqua, and Thiebaut De Schotten 2013; Jumah and Dossani 2024). Although gray matter atrophy of the amygdala and hippocampus in SD is well-documented (Boxer et al. 2003; Brambati et al. 2009; Chan et al. 2001; Collins et al. 2017; Hodges and Patterson 2007, 2; Marshall et al. 2018; Mummery et al. 2000; Rosen et al. 2002), limitations of conventional diffusion MRI have hampered investigation of their white-matter connections. FBA now pinpoints microstructural loss in these small tracts—changes largely missed by DTI/TBSS—thereby demonstrating their involvement in SD. The UF, running from the anterior temporal lobe to orbitofrontal cortex, integrates visceral–emotional cues with semantic memory and decision-making; its degeneration aligns with the combined semantic and socio-emotional deficits observed in SD (Agosta et al. 2010; Catani, Dell’Acqua, and Thiebaut De Schotten 2013). The cingulum bundle links medial temporal, limbic, and medial frontal regions; our finding of reduced fiber density, together with earlier reports of selective von Economo neuron loss in the anterior cingulate (Lam et al. 2014; Tan et al. 2014), helps account for the behavioral and affective changes typical of SD (Landin-Romero et al. 2016). The fornix, the principal efferent of the hippocampus, showed lowered fiber density in our cohort, echoing prior reports that disruption of the fornix predicts memory and emotional impairments in SD and underscoring FBA’s value for detecting limbic micro-degeneration (Elahi et al. 2017; Hodgetts et al. 2017; Modi et al. 2013).

Taken together, these limbic alterations demonstrate that SD pathology extends well beyond the anterior temporal cortex, undermining a broader network essential for memory, emotion, and behavioral regulation. Ultra-high-field diffusion imaging, combined with tract-specific functional and behavioral measures, will be crucial for clarifying how limbic disconnection shapes disease trajectory and for identifying targets for neuromodulatory or behavioral interventions aimed at preserving residual connectivity.

### Major white matter bundle involvement in semantic dementia

Previous diffusion tensor studies have consistently demonstrated degeneration of ATL projection tracts in SD – most consistently the ILF, UF, IFOF, and AF (Acosta-Cabronero et al. 2011; Agosta et al. 2010, 2015; Galantucci et al. 2011; Mahoney et al. 2013; Mandelli et al. 2014; Tu et al. 2016; Whitwell et al. 2010). Our combined TBSS and FBA approaches confirm these findings, with the most pronounced abnormalities in the hemisphere showing greater gray-matter loss. As in earlier work, RD proved more sensitive than FA for detecting these changes (Acosta-Cabronero et al. 2011; Agosta et al. 2010; Mahoney et al. 2013; Schwindt et al. 2013). Functionally, the ILF links the ATL to occipital-temporal visual regions that support object recognition; its disruption correlates with naming deficits, impaired semantic access and difficulties recognizing faces and emotions (Catani and Thiebaut de Schotten 2008; Herbet, Zemmoura, and Duffau 2018). The IFOF supports multimodal semantic control; left-hemisphere damage is associated with verbal semantic deficits, whereas right-hemisphere damage is linked to socioemotional impairments (Gonzalez Alam et al. 2024; de Zubicaray, Rose, and McMahon 2011). The UF connects the ATL to orbitofrontal cortex, bridging semantic and limbic networks; left-UF injury is linked to language-semantic deficits, while right-UF injury more often affects socio-emotional behavior (Oishi et al. 2015; Toller et al. 2022; Von Der Heide et al. 2013). Finally, AF abnormalities extend into inferior-parietal territory, indicating that white-matter damage may precede overt cortical thinning in these regions and highlighting the need to monitor dorsal as well as ventral pathways longitudinally. Taken together, degeneration of these tracts helps explain the heterogeneous clinical presentations of semantic dementia. Mapping their differential vulnerability provides a framework for tracking disease progression and for targeting future therapeutic or neuromodulatory interventions to specific white-matter pathways.

### Limitations

Despite its strengths, this study has some limitations. First, the sample size was modest because we restricted inclusion to patients in the early disease stage who also had multi-shell diffusion imaging data. While this strategy enabled precise characterization of early pathology, it inevitably reduced statistical power and may limit the generalizability of our findings. Second, registration or normalization errors during FBA processing could bias fiber-cross-section estimates—especially in regions of pronounced atrophy, where accurately aligning white-matter structures is intrinsically difficult. Nevertheless, the mirrored results obtained in left- and right-predominant ATL groups argue against major misalignment artefacts. Finally, in severely atrophic areas it remains challenging to disentangle gray- and white-matter damage, calling for cautious interpretation of tract-based metrics. Collectively, these limitations highlight the need for larger cohorts, further refinements to FBA registration pipelines, and rigorous quality-control procedures in future work.

### Conclusion and future directions

Advanced diffusion imaging adds crucial anatomical detail to our understanding of white-matter involvement in semantic dementia. Traditional skeleton-based methods such as TBSS capture the broad pattern of degeneration but miss many subtle changes because they cannot resolve crossing fibers or very small tracts. Fixel-based analysis (FBA) overcomes these limitations, detecting both microstructural loss (reduced fiber density) and macrostructural atrophy (reduced fiber cross-section) in pathways that are difficult to characterize with conventional techniques—most notably the anterior commissure and mesial-temporal projections to the amygdala and hippocampus—while also refining our view of established ATL-connected tracts.

Larger, longitudinal cohorts are now needed to test whether early FBA abnormalities precede—or merely accompany—the spread of cortical pathology and whether they predict distinct clinical phenotypes. Such work will determine the prognostic value of tract-specific metrics and help explain the heterogeneity of semantic dementia.

Finally, high-resolution diffusion metrics could guide therapeutic strategies. By pinpointing residual connectivity, they may inform the placement or targeting of neuromodulation approaches such as transcranial magnetic or direct-current stimulation and aid in monitoring treatment response. Integrating sophisticated imaging with personalized interventions therefore holds considerable promise for improving clinical outcomes and for clarifying the neurobiological mechanisms that drive this disease.

## Supplementary Files

This is a list of supplementary files associated with this preprint. Click to download.

- Fig1SM.tiff
- Table1.docx

## Data Availability

Upon publication, data from this study will be made available through an access-controlled repository within the FAIR Alzheimer’s Disease Data Initiative AD Workbench (https://fair.addi.ad-datainitiative.org). Requests for access can be submitted through the UCSF-MAC Resource Portal (Request form: http://memory.ucsf.edu/resources/data). Access will be granted following UCSF-regulated procedures in accordance with ethical guidelines for the reuse of sensitive data. Researchers seeking access must submit a Material Transfer Agreement, which is available at: https://icd.ucsf.edu/material-transfer-and-data-agreements.

## Abbreviations

WM: white matter
dMRI: diffusion magnetic resonance imaging
DTI: diffusion-tensor imaging
TBSS: tract-based spatial statistics
FBM: Fixel-based morphometry
FBA: Fixel-based analysis
FWE: family-wise error
FA: fractional anisotropy
MD: mean diffusivity
AD: axial diffusivity
RD: radial diffusivity
FD: fiber density
FC: fiber-bundle cross-section
FDC: Fixel Density Cross-Section
SD: semantic dementia
svPPA: semantic variant primary progressive aphasia
sbvFTD: semantic behavioral fronto-temporal dementia
MMSE: Mini-Mental State Examination
CDR: Clinical Dementia Rating
ATL: anterior temporal lobe
ILF: inferior longitudinal fasciculus
UF: the uncinate fasciculus
IFOF: inferior frontal occipital fasciculus
AF: Arcuate fasciculus
CC: corpus callosum
MPRAGE: Magnetization-prepared rapid acquisition with gradient echo acquisition
TIV: total intracranial volume
